# Secretory Vesicles Deliver Cdc42p to Sites of Polarized Growth in *S. cerevisiae*

**DOI:** 10.1371/journal.pone.0099494

**Published:** 2014-06-19

**Authors:** Shubha A. Dighe, Keith G. Kozminski

**Affiliations:** 1 Department of Biology, University of Virginia, Charlottesville, Virginia, United States of America; 2 Department of Cell Biology, University of Virginia, Charlottesville, Virginia, United States of America; Institute of Biology Valrose, France

## Abstract

The activation and localization of the Rho-family GTPase Cdc42p at one pole of a cell is necessary for maintaining an axis of polarized growth in many animal and fungal cells. How the asymmetric distribution of this key regulator of polarized morphogenesis is maintained is not fully understood, though divergent models have emerged from a congruence of multiple studies, including one that posits a role for polarized secretion. Here we show with *S. cerevisiae* that Cdc42p associates with secretory vesicles *in vivo*.

## Introduction

How a cell establishes and maintains an axis of polarized growth is a key question in developmental biology. Failure of this process can result in aberrant cell division as well as developmental abnormalities [1], [2]. Inevitably there are species-specific and cell type-specific differences in the mechanics of this process, but commonalities do exist [3], [4]. In animals and fungi, for example, the monomeric Rho-family GTPase Cdc42p is essential for establishing and maintaining an axis of polarized growth in many cell types [5], [6]. In the budding yeast *S. cerevisiae*, Cdc42p must associate with and be transiently activated at a single site on the plasma membrane to establish an axis of polarized growth that leads to bud formation [7]–[9]. Although not required for the establishment of the axis of growth, which appears to utilize a Turing reaction-diffusion mechanism [8], polarized secretion and endocytic recycling appear necessary for maintaining a cap of Cdc42p at the bud tip of *S. cerevisiae* [9]–[12]. Some observations however challenge the necessity of polarized secretion in this process [13].

How polarized secretion contributes to the maintenance of a Cdc42p cap at the bud tip is not known. One model posits that secretory vesicles deliver Cdc42p to the bud tip, the site of polarized growth [10]. Another, based on computational modeling, posits that secretion is insufficient to maintain a sufficient concentration of activated Cdc42p at the bud tip and that secretory vesicles may deliver to the bud tip, not Cdc42p, but another factor necessary for maintaining Cdc42p-dependent bud growth [14], [15]. The validity of the first model depends upon evidence that Cdc42p associates with secretory vesicles. To date, evidence of vesicle association has rested on *in vitro* [16]–[19] or orthologous *in vivo* observations. For example, a GFP-labeling study with astrocytes showed Cdc42p associated with vesicles [20]. Because it is known that GFP-tagging interferes with Cdc42p function *in vivo* [21], most likely due to the proximity of the fluorescent moiety to the effector domain of the GTPase, and because bud geometry makes it difficult to resolve individual vesicles at the bud tip by light microscopy, we combined morphometric analysis with immunogold labeling to determine whether Cdc42p associates with *S. cerevisiae* secretory vesicles *in vivo*. Our EM-level analysis revealed this association, which is further confirmed by a complementary cell fractionation scheme in which Cdc42p was identified on immuno-isolated secretory vesicles.

## Results & Discussion

### Cdc42p Localizes to Secretory Vesicles *In vivo*

**Figure 1:**
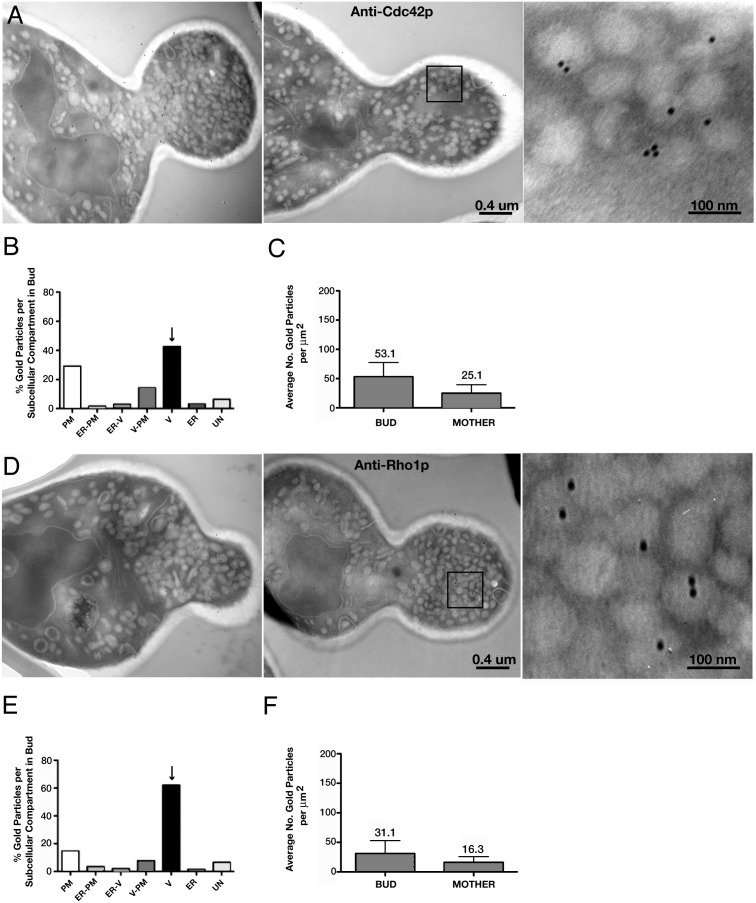
EM immunogold localization of Cdc42p in *sec6-4^ts^* cells. EM immunogold localization of Cdc42p (A) or Rho1p (D) in log-phase *sec6-4* cells shifted to 37°C for 60 min. B and E) Distribution of gold particles among various membrane compartments within buds of the same preparations shown in (A) and (D) when probed for Cdc42p (B; n = 37 cells) or Rho1p (E; n = 33 cells). PM, plasma membrane; V, 80–100 nm diameter vesicles; ER, endoplasmic reticulum; ER-PM, endoplasmic reticulum and/or plasma membrane; ER-V, endoplasmic reticulum and/or vesicles; V-PM, vesicles and/or plasma membrane; UN (unassigned), membrane compartment of ambiguous identity. C and F) Average number of gold particles within the bud and mother of the same cell, scored on the same section, standardized to an area of 1 um^2^. Error bars indicate SEM. Cells and sections scored in (C and F) are the same as those scored in (B and E), respectively.

Several studies have suggested that the small Rho-family GTPase Cdc42p associates with secretory vesicles in *S. cerevisiae* [17], [19], [22]. To conclusively demonstrate the *in vivo* occurrence of this association, we prepared EM thin sections of whole cells for immunogold labeling, probing them with an antibody that binds Cdc42p (Figure 1A). We found Cdc42p associated with the plasma membrane, as expected from previous localization studies with light and electron microscopy [22], [23], as well as with vesicles of 80–100 nm diameter, the signature size of post-Golgi secretory vesicles in *S. cerevisiae* [24], [25]. Because only a few secretory vesicles are observable per section of wild-type cells [24], [25], we initiated our studies with *sec6-4^ts^* cells. These cells have a temperature-sensitive defect in the exocyst complex subunit Sec6p that results in a rapid accumulation secretory vesicles within the bud upon shift to 37°C [26]. With immunogold labeled thin sections of *sec6-4^ts^* cells grown at 37°C for 60 min, we determined (see Materials and Methods) whether Cdc42p localized to one of the following membrane compartments in bud; plasma membrane, 80–100 nm diameter vesicles, or endoplasmic reticulum. When more than one of these membrane compartments were found within a 30 nm radius of the gold particle, the extended length of an antibody (1° and 2°)-gold complex [27], [28], Cdc42p was categorized as plasma membrane/ER-, vesicle/ER-, or vesicle/plasma membrane-associated, even though in actuality Cdc42p may only be associated with one membrane in each category pair. More than 40% of the gold particles (n = 490; 37 cells) were scored as associated with secretory vesicles that have a diameter of 80–100 nm (Figure 1B). Significant gold labeling of the plasma membrane was also observed, although no clustering of the label was observed at the bud tip. The inability to detect a cap of Cdc42p at the bud tip by this method was mostly likely due to epitope inaccessibility in the absence of ionic detergent [23]. Together these data indicate that Cdc42p associates with secretory vesicles as well as with the plasma membrane *in vivo* in *S. cerevisiae*.

We ascertained the specificity of gold particle labeling with three analyses. First, we asked whether the distribution of gold particles across a section of a cell was uniform. For each cell in our analysis, we measured the areas of the mother cell and bud. The numbers of gold particles in each area were then counted and standardized to a calculated area of 1 um^2^. Exclusive of the cell wall, buds had, on average, twice as many gold particles as mothers of the same cell (53.1 vs. 25.1/um^2^; p<0.001, Student’s t-test; Figure 1C). Therefore, gold particle labeling of the sections was non-uniform, indicating specific labeling. A uniform deposition of antibody-conjugated gold particles would have indicated non-specific labeling. Second, we asked whether any gold labeling was the result of non-specific labeling by the secondary antibody. No gold labeling was found in the absence of the primary antibody, indicating that labeling is dependent upon the anti-Cdc42p primary antibody (data not shown). Third, sections of *sec6-4^ts^* cells grown at 37°C for 60 min labeled with an antibody against the small Rho-family GTPase Rho1p gave similar, yet different results (Figure 1D). In this case, more than 60% of the gold particles (n = 198 gold particles; 33 cells) were scored as associated with 80–100 nm vesicles (Figure 1E). As observed with the anti-Cdc42p antibody, buds and mother cells showed a significant difference (p<0.002; Student’s t-test) in gold labeling, with an average of 31.1 gold particles/um^2^ in buds versus 16.3 gold particles/um^2^ in mother cells (Figure 1F). Although previous studies established the association of Rho1p with secretory vesicles in yeasts [16]–[18], [29], these results show the first immunogold localization of Rho1p to 80–100 nm vesicles in any yeast, including *S. cerevisiae*. Taken together, these data indicate that the immunogold labeling in our studies is antigen-specific and antigen-dependent.

**Figure 2:**
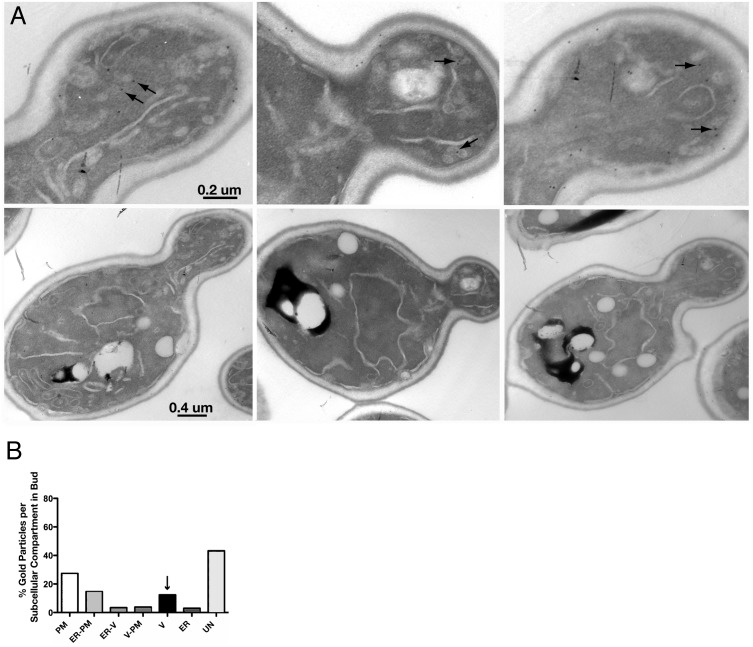
EM immunogold localization of Cdc42p in wild-type cells. A) EM immunogold localization of Cdc42p in log-phase wild-type cells (DDY904) grown at 25°C. Panels in top row show the buds of the corresponding cells in the bottom row. Arrows indicate gold labeling of secretory vesicles. B) Distribution of gold particles among various membrane compartments within buds of the same preparations shown in (A; n = 36 cells). Membrane compartment categories are the same as described for Figure 1B.

The association of Cdc42p with secretory vesicles occurs in wild-type cells. To exclude the possibilities that Cdc42p association with secretory vesicles occurs only in *sec6-4^ts^* cells or during cell growth at 37°C, we probed thin sections of wild-type cells grown at 25°C for Cdc42p (Figure 2A). We found gold labeling of 80–100 nm vesicles, in addition to the plasma membrane and ER. In sections of wild-type cells, approximately 10% of the gold particles scored labeled 80–100 nm vesicles (n = 1050 gold particles; 36 cells), as compared to >40% with *sec6-4^ts^* cells grown at 37°C (Figure 2B). This difference in labeling is consistent with fewer 80–100 nm vesicles per section of wild-type cells versus *sec6-4^ts^* cells. We also found that labeling of the plasma membrane showed little variance between wild-type and *sec6-4^ts^* cells, indicating that any change in labeling is specific to secretory vesicles. As was observed with sections of *sec6-4^ts^* cells grown at 37°C, approximately 30% of the gold particles scored labeled the plasma membrane of wild-type cells grown at 25°C. Also, as before, a significant difference in the average amount of labeling (77.2 vs. 36.5 gold particles/um^2^; p<0.001, Student’s t-test) was seen in buds and mother cells, respectively. Therefore, association of Cdc42p with vesicles *in vivo* is neither strain nor temperature-dependent.

### Cdc42p Co-fractionates with Immuno-purified Secretory Vesicles

To further validate that Cdc42p associates with secretory vesicles in *S. cerevisiae*, we probed immuno-isolated secretory vesicles for Cdc42p with the same antibody used for our immuno-localization experiments.

**Figure 3:**
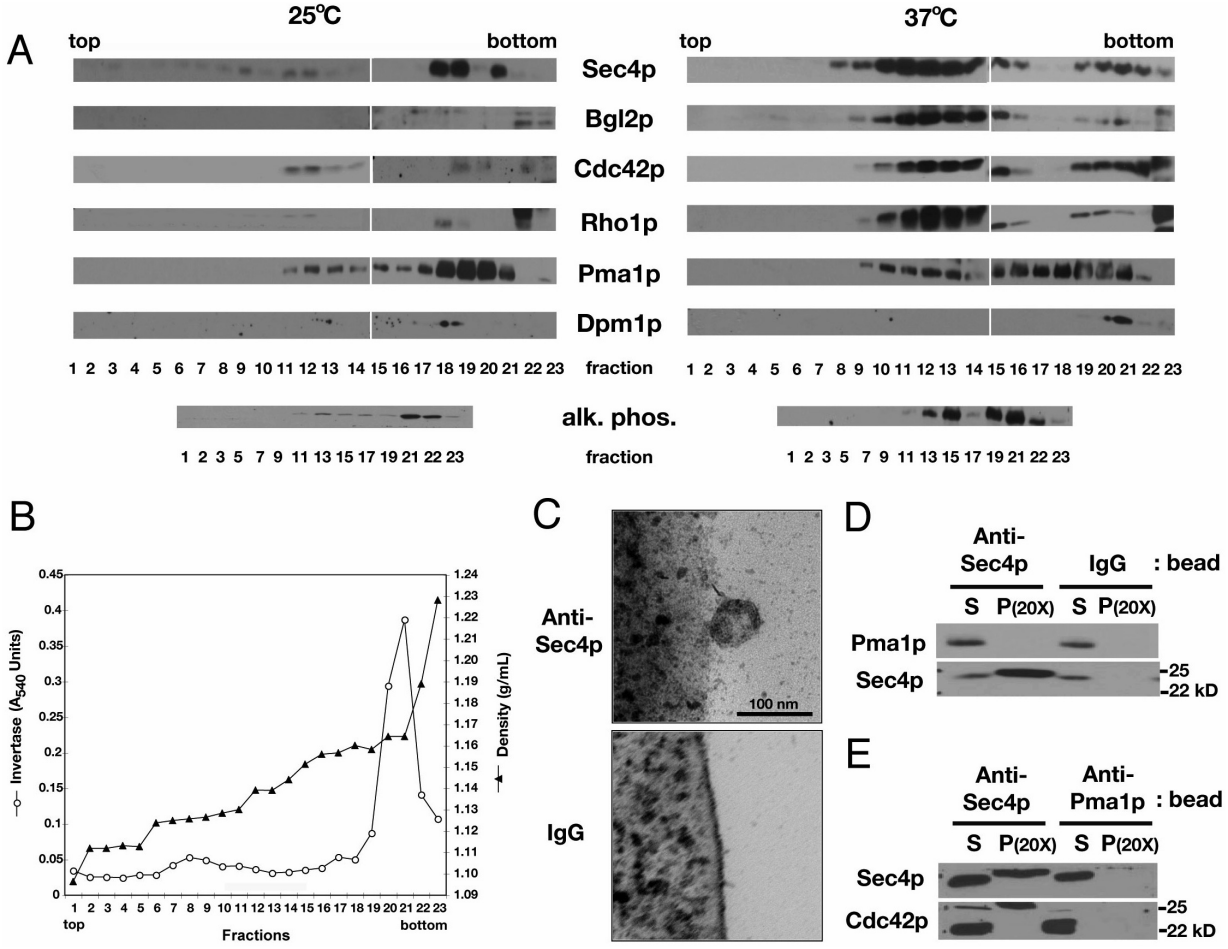
Co-purification of Cdc42p with low density, Sec4p-marked vesicles. A) Immunoblots of 100,000×*g* pellets of fractions collected from an 18–34% Nycodenz-sorbitol buoyant density gradient loaded with extracts prepared from equivalent OD_600_ units of *sec6-4^ts^* cells grown at 25°C or 37°C for 90 min. Each lane was loaded equivalent fraction volumes and, for the same probe, incubated with antibody of equivalent titer for the same amount of time. All blots are from the same fractionation, with the Sec4p and Bgl2p panels reprinted with permission from Alfaro *et al*. [37] © 2011 *Traffic*. All blots at an indicated temperature were prepared from the same fractions from the same gradient. B) Graph showing invertase activity across “37°C” buoyant density gradient shown in (A) and sucrose density across an unloaded gradient processed in parallel. Fraction densities were calculated based on a standard curve of Nycodenz-sorbitol concentration versus refractive index. C) Thin section electron micrographs showing vesicle association with a M500 Dynabead coated with mouse monoclonal anti-Sec4p antibody (left), but not with the same type of bead coated with mouse IgG (right), after incubation with a pooled, Sec4p-enriched, buoyant-density gradient fraction from *sec6-4^ts^* cells grown at 37°C for 90 min (comparable to fractions 11–14 in (A)). D) Immunoblots of a pooled, Sec4p-enriched, low-density gradient fraction (S), after incubation with Protein G-Dynabeads (P) coated with a monoclonal antibody against Sec4p or mouse IgG, and probed for plasma membrane marker Pma1p or post-Golgi secretory vesicle marker Sec4p. E) Immunoblots of pooled, Sec4p-enriched, low-density gradient fraction (S), after incubation with Dynabeads coated with a monoclonal antibody against Sec4p or Pma1p (P), probed for Cdc42p or Sec4p. In (D) and (E), the stoichiometric ratio of pellet to supernatant is 20∶1.

Secretory vesicles were isolated in a two-step procedure from *sec6-4^ts^* cells cultured at 37°C. In the first step, membranes and membrane compartments were separated on a Nycodenz-sorbitol buoyant density gradient (Figure 3A). Cdc42p was found in two distinct peaks (fractions no. 11–15 and 19–22) at the reported densities of Bgl2p- and invertase (Suc2p)-marked secretory vesicles, 1.14 g/mL and 1.165 g/mL, respectively (Figures 3A, B; [30]). Immunoblotting also confirmed the presence of Bgl2p in the fractions of lower density; whereas, an assay for invertase activity confirmed the presence of invertase in the fractions of higher density (Figure 3B). A similar distribution across the density gradient was observed for the Rho-family GTPase Rho1p and Rab-family GTPase Sec4p, the latter of which is a well-established marker for post-Golgi secretory vesicles in *S. cerevisiae* [31]. An increase in the amount of Cdc42p, Rho1p, Sec4p, and Bgl2p, especially in low-density fractions (no. 11–15), correlated with an increased number of secretory vesicles per cell at 37°C versus 25°C (compare Figures 1A and 2A). That is, low-density membrane fractions of cells grown at 25°C, for example, showed substantially less Cdc42p and Sec4p (and undetectable amounts of Bgl2p and Rho1p) than found in the same fractions of cells grown at 37°C (Figure 3A). In comparison to markers for other membrane compartments, the peaks of Cdc42p, Rho1p, Sec4p, and Bgl2p showed varying degrees of overlap with the plasma membrane marker Pma1p, a H^+^-ATPase, and the vacuole marker alkaline phosphatase, but not with the ER marker Dpm1p, dolichol phosphate mannose synthase [32]. Taken together, these data indicate an effective separation of secretory vesicle populations, but not necessarily membrane or membrane compartment homogeneity within a given fraction. For this reason, we added a second step to our vesicle isolation procedure, which involved using antibody-coated Dynabeads to isolate secretory vesicles from gradient fractions, enriched for the secretory vesicle markers Sec4p and Bgl2p. We focused on this vesicle population, rather than on vesicles marked by Sec4p/invertase, because Sec4p/Bgl2p-marked vesicles support Cdc42p-dependent polarized bud growth [33].

We found that anti-Sec4p Dynabeads can be used to isolate 80–100 nm vesicles. We chose an antibody against Sec4p as an affinity matrix because Sec4p is present on the cytoplasmic surface of vesicles [31]; whereas, Bgl2p is luminal [34]. From pooled gradient fractions enriched in Sec4p, Bgl2p, Cdc42p, we were able to isolate 80–100 nm vesicles with anti-Sec4p coated Dynabeads, but not with IgG-coated Dynabeads (Figure 3C). Consistent with this observation, immunoblots showed that Sec4p, but not the plasma membrane marker Pma1p, co-fractionated with anti-Sec4p Dynabeads (Figure 3D). Neither Sec4p nor Pma1p co-fractionated with Dynabeads coated with IgG. These data demonstrate that the interaction of secretory vesicles with the beads is specific.

More significantly, we found that Cdc42p, along with Sec4p, co-fractionates with anti-Sec4p Dynabeads after the beads are mixed with a pooled buoyant density gradient fraction enriched in Sec4p and Cdc42p (Figure 3E). This too is a specific interaction, considering that neither Cdc42p nor Sec4p co-fractionates with anti-Pma1p coated beads. We also noted that the molecular weights of Sec4p and Cdc42p, when co-fractionated with the Dynabeads, were slightly larger than predicted and larger than those of the same GTPases that remain in the supernatant. The reason for these small increases in molecular weight is not known, though it is possible these shifts in molecular weight represent a pattern of post-translational modifications unique to the association of these GTPases with secretory vesicles. Taken together, these *in vitro* data support our *in vivo* observation that Cdc42p associates with secretory vesicles in *S. cerevisiae*.

The functional significance of the association of Cdc42p with secretory vesicles *in vivo* is not entirely clear. Although our results indicate that secretory vesicles can serve as a vehicle for the delivery of Cdc42p to sites of polarized growth, in support of a model by Wedlich-Soldner *et al*. [10], it is not clear to what extent, if any, this delivery contributes to the maintenance of an axis of polarized growth supported by exocytosis. Modeling by Layton *et al*. [14] and Savage *et al*. [15] proposes that vesicles and their trafficking do not have the capacity to bring Cdc42p to the bud tip in amounts sufficient to maintain a cap of Cdc42p at the bud tip in support of an axis of polarized exocytosis. To constrain these opposing models, quantification of the amount of Cdc42p associated with individual secretory vesicles will be needed as well as a determination of how much Cdc42p on vesicles is recycled from the plasma membrane versus how much is newly synthesized and making its first vesicle-associated journey to the plasma membrane. Alternatively, Cdc42p may associate with secretory vesicles, not as a passenger to the plasma membrane, but to regulate a vesicle-associated activity.

## Materials and Methods

### Yeast Strains and Reagents

Rich medium (YPD) containing 1% Bacto-yeast extract, 2% Bacto-peptone, and 2% glucose was used for growing NY17, *sec6-4 ura3-52* [24] and DDY904, *his3Δ200 lys2-801 ura3-52 leu2-3, 112* [23] at 25°C or 37°C. BD Laboratories, Inc. **(**Sparks, MD**)** supplied the reagents for media.

### Immunogold Label Electron Microscopy

Cells were cultured in rich medium (YPD) at 25°C to mid log phase (∼0.25 O.D._600_), then split between two flasks incubated at 25 or 37°C for 1 h. To 10 O.D. units of log-phase culture in 50 mL conical tubes, 2X buffered-fixative (1 M D-sorbitol, 8% (v/v) paraformaldehyde (18505, Ted Pella, Inc., Redding, CA), 0.8% (v/v) glutaraldehyde (18426, Ted Pella Inc., Redding, CA), 160 mM potassium phosphate buffer, pH 6.7) was rapidly added, starting a 60 min room temperature incubation. Fixed cells were then harvested by centrifugation at 1500×*g* for 8 min at room temperature. Pellets were washed gently with 10 mL of sorbitol-phosphate buffer (0.5 M sorbitol in 80 mM potassium phosphate buffer, pH 6.7), followed by centrifugation at 1500×*g* for 3 min. Pellets were resuspended as before, though in 5 mL 80 mM potassium phosphate buffer containing 0.25 M sorbitol. After resuspension, cells were transferred to a glass test tube and centrifuged as above. Pellets were then resuspended in 5 mL of 1% (w/v) NaIO_4_ for 10 min at room temperature, centrifuged at 1500×*g* for 3 min, washed with deionized water, and centrifuged again. Pellets were then resuspended in 5 mL of 50 mM NH_4_Cl, centrifuged again, and resuspended in 3 mL 30% ethanol, preceding serial dehydration in 50, 70, 80, 85, 90, 95, 100, and 100% ethanol. Unless specified otherwise, all incubations were 5 min at room temperature, with gentle rocking. After dehydration, cells were gently pelleted and resuspended in 1.5 mL of 2∶1 ethanol: LR White resin (14383, Electron Microscopy Sciences, Hatfield, PA) and nutated for 1 h, followed by overnight incubation in 1∶1 ethanol: resin mixture. Cells were then gently pelleted using a fixed angle VS-15 microcentrifuge (Shelton Scientific, Iowa**)** and resuspended in 1 mL 1∶2 ethanol: resin and nutated 2 h, followed by two 2 h incubations in 100% LR White. Resin-infiltrated cells were then transferred to a size 4 gelatin capsule (07348, Polysciences, Warrington, PA), which was then nested in a size 00 gelatin capsule. The capsule was kept uncapped for 30 min to allow cells to settle before additional resin was added to completely fill the capsule. To harden the resin, capsules were baked at 45°C for 24 h.

After the embedding, ∼85 nm sections were cut with a diamond knife and placed on 200 mesh nickel grids. Sections were hydrated with PBS and then incubated in 10 mM glycine (in PBS) for 10 min, followed by 15 min incubation with Blocking Solution (25596, Electron Microscopy Sciences, Hatfield, PA). The sections were then incubated overnight at 4°C, in a moist chamber, in a 15 µL droplet of primary antibody (rabbit anti-Rho1 [35] or anti-Cdc42 peptide [23]) diluted 1∶10 in Incubation Buffer (IB; PBS+ 0.2% (v/v**)** Aurion BSA-C (# 25557, Electron Microscopy Sciences, Hatfield, PA), followed by three successive 10 min washes at room temperature with 0.1% Tween 20 (25564, Electron Microscopy Sciences, Hatfield, PA) diluted in IB. Sections were then incubated at room temperature for 1 h with secondary antibody, goat anti-rabbit IgG, conjugated to 10 nm gold particles (25109, Electron Microscopy Sciences, Hatfield, PA) diluted 1∶50 in IB, followed by three successive washes with 0.1% Tween 20 in IB as described above. A final wash used deionized water. After the final wash, grids were wicked dry with Whatman filter paper. For contrast, sections were stained with 2% (w/v) aqueous uranyl acetate (22400, Electron Microscopy Sciences, Hatfield, PA) for 4 min at room temperature, followed by 3 quick rinses with deionized water and dried as before.

After air-drying the grids, sections were viewed with a JEOL 1230 electron microscope and images were captured using a SIA-12C, 4 K×4 K digital camera at 20 or 40 K magnification. In captured images of thin sections, areas within cells and radii around individual gold particles were demarcated and determined with ImageJ software (v.1.38, NIH). The method of Boyd *et al*. [36] was used to score a particular protein as “associated” with a particular membrane compartment. Membrane compartments were identified visually. Only gold particles located on or within 30 nm of the plasma membrane were scored. The narrowest width of the bud neck was chosen to demarcate the boundary between mother and bud.

### Isolation of Secretory Vesicles

Cell fractions enriched in secretory vesicles or other membrane compartments were obtained on Nycodenz-sorbitol buoyant density gradients as described previously in Alfaro *et al*. [37]. The distribution of membrane compartments across the gradient was determined principally by immunoblotting [38] for compartment specific markers, following SDS-PAGE. Blots were probed with mouse anti-Sec4p (1∶10,000; mAb1.2.3, gift of Dr. Patrick Brennwald, University of North Carolina-Chapel Hill), rabbit anti-Bgl2 (1∶7500; [37]), rabbit anti-Cdc42 peptide (1∶1000, [23]), rabbit anti-Rho1 peptide (1∶500; [34]), mouse anti-Pma1p (1∶10,000; ab4645, Abcam, Cambridge, MA), mouse anti-Dpm1 (1∶1000; A6429, Molecular Probes, Inc., Eugene, OR), mouse anti-alkaline phosphatase (1∶1000; A6458, Life Technologies/Molecular Probes, Eugene, OR). For ECL, horseradish peroxidase-linked rabbit IgG (NA934, GE Healthcare, Piscataway, NJ) or mouse IgG (NA931, GE Healthcare, Piscataway, NJ) were used at 1∶5000 for 30 min at room temperature. To identify fractions enriched in Suc2p (invertase)-marked vesicles, fractions were diluted 1∶10 with TE (10 mM Tris-Cl, 1 mM EDTA, pH 7.5) and assayed for invertase activity as described in Dighe and Kozminski [39].

To increase the homogeneity of vesicle preparations, vesicles were immuno-isolated from buoyant density gradient fractions with monoclonal antibody against Sec4p (mAb1.2.3) bound to Protein G-Dynabeads (100-03D, Invitrogen, Oslo, Norway). Monoclonal antibody Pma1p (40B7) (ab4645, Abcam, Cambridge, MA**)** and mouse IgG, (61-5600, Invitrogen, CA) served as controls for binding specificity. To bind antibodies to beads, 50 µL Dynabeads, washed thrice with 250 µL ice-cold PBS, were mixed with 1 µL antibody diluted in 250 µL PBS. After incubation at 25°C for 20 min with rotation, the beads were washed twice with PBS, once with PBS containing 1 mg/mL BSA, followed by two more washes with PBS. The beads were then mixed with 400 µL of a pooled density gradient fraction (400 µL each of fractions 9–15 were mixed to form a “pooled” fraction), which was diluted further by addition of 600 µL Tris-sorbitol buffer (50 mM Tris-Cl, 1 mM EDTA, 0.8 M Sorbitol, pH 7.5). Rotational mixing for 2 h at 4°C followed. Dynabeads were then concentrated with a magnet. Supernatants were mixed 1∶1 with 4X Sample Buffer [37]. The beads were washed thrice with Tris-sorbitol buffer and resuspended in 2X Sample Buffer. Polypeptides in each fraction were separated by SDS-PAGE and analyzed by immunoblotting as described above.

### Thin Section Electron Microscopy of Immuno-isolated Vesicles

For visualization by thin section electron microscopy, vesicles were immuno-isolated with M500 Dynabeads, favored for the image quality of their highly even surfaces. Sec4p and IgG antibodies (same as above) were bound, as described above, to 20 µL M-500 beads. The antibody-bound beads were mixed for 2 h, at 4°C, with pooled buoyant density gradient fractions that contain exocytic vesicles, followed by three washes of 250 µL Tris-sorbitol buffer. The beads were then washed with 40 mM potassium phosphate buffer, pH 7.2 containing 0.8 M sorbitol, followed by fixation at room temperature for 1 h using the same phosphate-sorbitol buffer though containing 3% (v/v) paraformaldehyde (18505, Ted Pella, Inc., Redding, CA) and 1% (v/v) glutaraldehyde (18426, Ted Pella Inc., Redding, CA). Following fixation, the beads were washed twice with 1 mL phosphate-sorbitol buffer and then mixed with 1 mL cold 40 mM potassium phosphate buffer, pH 7.2 containing 0.5% (v/v) osmium tetroxide and 0.8% (w/v) potassium ferricyanide. Beads were then incubated on ice for 1 h, washed 3 times with 1 mL EM-grade deionized water, and finally mixed with 1 mL of 0.5% (w/v) uranyl acetate solution prior to incubation at 4°C, overnight, in the dark. Beads were then pelleted using a magnet and washed twice with 1 mL deionized water. The samples were then dehydrated with increasing concentrations of ethanol; 1 mL 5% ethanol for 30 min, then 10, 20, 40, 80 and 100% ethanol for 15 min each, with the final wash made with 0.5 mL Spurr’s resin (14300, Electron Microscopy Sciences, Hatfield, PA) prepared in ethanol. All these and subsequent incubations were made at room temperature. Dehydrated beads were then mixed gently with 0.5 mL 5% Spurr’s resin for 2 h, followed by 20% resin overnight. The next day, beads were mixed with 0.4 mL 33% resin for 6 h and then overnight in 0.4 mL 50% resin. The following day, the beads were mixed with 0.4 mL 66% resin and nutated for 4 h, with a final overnight incubation with 100% gently layered on the beads. Resin was then decanted and 0.4 mL 100% resin added for 5 h. To polymerize the resin, the tubes were heated at 70°C for at least 2 d. After the embedding, ∼85 nm sections were cut with a diamond knife and placed on 200 mesh copper grids. Staining was done with lead citrate and 3% uranyl acetate. After air-drying the grids, sections were viewed as described above.
